# Resiliency in Child–Caregiver Dyads and the Impact on Health Outcomes in Sickle Cell Disease

**DOI:** 10.3390/children12040394

**Published:** 2025-03-21

**Authors:** Jessica A. Zavadil, Melissa Azul, Brian D. Carpenter, Cecelia Calhoun

**Affiliations:** 1 Department of Pediatrics, Indiana University, Indianapolis, IN 46202, USA; 2 Department of Psychological and Brain Sciences, Washington University, St. Louis, MO 63103, USA; bcarpenter@wustl.edu (B.D.C.); cece.calhoun@yale.edu (C.C.); 3 Department of Pediatrics, Medical College of Wisconsin, Milwaukee, WI 53226, USA; mazul@mcw.edu; 4 Department of Internal Medicine (Hematology), Yale University, New Haven, CT 06510, USA

**Keywords:** resiliency, sickle cell disease, stress, COVID-19

## Abstract

**Background/Objectives**: Resiliency is critical in coping with stressors associated with chronic health diseases. Sickle cell disease (SCD) is a chronic blood disorder in which familial psychosocial functioning impacts disease outcomes. We hypothesized that caregiver perceived stress and resiliency are related to the resiliency of children with SCD and may influence SCD clinical outcomes. **Methods**: Child–caregiver dyads completed the Perceived Stress Scale (PSS-10), Connor Davidson-Resilience Scale (CD-RISC), and used a 1–5 Likert scale to rate the frequency of stressors they experience, including the COVID-19 pandemic. **Results**: Of the 55 child participants, 36% reported a history of stroke, 7% a bone marrow transplant, and 25% frequent (≥3) emergency room visits within last year. Dyad median resiliency scores (68.5 vs. 75.8) and stress scores (16.1 vs. 15.3) were similar and consistent with population studies. Child resiliency was not associated with child (*r* = −0.21, *p* = 0.12) or caregiver (*r* = −0.16, *p* = 0.26) perceived stress. Caregiver and child resiliencies had a significant positive correlation (*r* = 0.38, *p* = 0.0046) but no relationship across dyads with perceived stress scores. Children with one to two hospitalizations within the last year had significantly lower median resiliency scores compared with those who had experienced no hospitalizations (median 65 vs. 76, *p* = 0.0386), but displayed no relationship with genotype, history of stroke, or stem cell transplant. During the COVID-19 pandemic, both groups rated “worry about my/my child’s sickle cell disease” as the most frequent psychosocial stressor. **Conclusions**: In a cross-sectional cohort study that explored the relationship between caregiver resiliency and child resiliency in SCD, we found that caregiver resiliency and child resiliency were strongly correlated, while child resiliency showed no significant association with perceived stress. Higher child resiliency scores were associated with fewer hospitalizations. The results indicate the need for interventions to increase both child and caregiver resiliency in SCD, as it may contribute to health outcomes in SCD. Further research is needed to explore cofounding factors influencing resiliency in children with SCD.

## 1. Introduction

Resiliency is a key adaptive response to stressors related to a person’s perceived ability to cope with stress and adversity. With adequate external support, such as a supportive caregiver, stressors can lead to the development of resiliency [1]. Without this support, stress becomes toxic and leads to the dysregulation of immune and neuroendocrine pathways [2]. Longitudinal studies have identified that adverse childhood experiences (ACEs) are associated with decreased resiliency and a dose-dependent increase in the prevalence of chronic illnesses including obesity, asthma, autism spectrum disorder, and attention deficit hyperactive disorder in children [3], and additionally depression, diabetes, stroke, chronic lung disease, rheumatoid arthritis, and heart disease in adults [4,5,6]. ACEs include exposure to violence, abuse (emotional, physical, or sexual), family discord or divorce, parental substance abuse, mental health problems, death, or incarceration, and social discrimination [3]. Consistent with the toxic stress model, there is evidence that stress from ACEs can contribute to disease pathophysiology, such as that of asthma [7]. Moreover, children with chronic illnesses are more likely to experience ACEs [3]. This complicates the circumstances faced by children with chronic illnesses, as they also navigate the unique challenges associated with the illnesses themselves, such as more frequent hospitalizations or physical or mental impairments.

Previous studies have shown that resiliency can mitigate the impact of ACEs, such as reducing grade retention [3]. Mindfulness training in schools and family-centered medical homes are protective factors and improve resiliency in children with high risk for ACEs [3]. Resiliency has also been associated with improved chronic disease management, such as that of diabetes [8,9]. Understanding the role of stressors and resiliency in children with chronic disease is a key component in preventing comorbidities and improving disease management.

Sickle cell disease (SCD) is another chronic illness where resiliency has been associated with improved outcomes in children. This inherited disease of the red blood cells is associated with significant childhood morbidities including stroke, vaso-occlusive crises, and avascular necrosis. These complications contribute to decreased quality-adjusted life years, income, and productivity and a >20-year reduction in life expectancy [10]. Previous studies have shown that comorbidities, like silent stroke, can affect children’s functioning and are associated with decreased school performance [11]. Similarly, higher ACE scores in children with SCD are associated with more emergency room (ER) visits and acute chest syndrome [12]. Fortunately, resiliency in children with SCD is associated with reduced pain [11], improved grade retention [13], and increased readiness for transition to adult medical care [14]. Identifying stressors and how they relate to resiliency in children with SCD may be an important factor for improving health outcomes.

Previous studies examining resiliency in children with SCD have focused on familial psychosocial functioning as it relates to SCD management and demonstrate the importance of social support. For example, one study showed the increased risk of psychosocial distress in children whose caregivers had less education or were divorced; when there were fewer adults or more children in the home; and in the presence of financial difficulties [15]. Studies in children with other chronic illnesses have shown similar relationships between child and caregiver distress and coping. For example, in parent–child dyads involving adolescents with cancer, higher caregiver distress was associated with decreased child resiliency [16]. In contrast, in children with chronic pain, increased caregiver psychological flexibility was associated with increases in child’s pain acceptance, pain self-efficacy, and quality of life [17]. However, the relationship between a child’s resiliency or perceived stress and that of their caregiver has not been explored in children with SCD. We hypothesized that, for children with SCD, perceived stress and resiliency are related to their caregiver’s perceived stress and resiliency. We also hypothesized that lower resiliency in either would be associated with hospitalization, history of transplant, and history of stroke. Secondarily, we sought to identify sources of stress for these families during the onset of the COVID-19 pandemic and the perceived impact on sickle cell disease management.

## 2. Materials and Methods

### 2.1. Participants

Study participants were recruited from St. Louis Children’s Hospital Hematology Clinic from June 2020 to January 2021. Eligible participants were identified by date of birth and diagnosis through chart review. Inclusion criteria were the child being ≥12 years old, being English speaking, and having a sickle cell disease diagnosis of any genotype (not including sickle cell trait). Exclusion criteria included the child being in foster care or having a sibling already enrolled. Caregiver was defined as the self-identified primary caretaker living with the child. Potential participants were approached by phone or in clinic. Flyers were provided in clinic with information about the study. Caregivers and children ≥18 years old provided consent and children <18 years old provided assent over the phone or in person in a private clinic room. Power analysis was conducted based on previous publication assessing resiliency in child–caregiver dyads [16], and we sought to recruit 73 pairs for a power of 0.80 with the alpha set at 0.05. The study design was approved by Washington University Institutional Review Board.

### 2.2. Study Participation

Once consent was obtained, participants were asked to complete surveys on Redcap online or using a printed version. Redcap surveys were sent by email with reminders every 4 weeks on up to 3 occasions. Participants who completed surveys by hand returned them to the study coordinator on the same day, and they were entered into Redcap then destroyed. Once study participation by both the child and caregiver was completed, a forty-dollar gift card was sent electronically or by mail to the caregiver.

### 2.3. Measures

In addition to providing demographic data, child and caregiver participants were asked to complete the Perceived Stress Scale (PSS-10) [18] and Connor Davidson-Resilience Scale (CD-RISC) [19] for themselves, score their sickle cell disease management on a 1–5 Likert scale (strongly disagree to strongly agree) before and during the COVID-19 pandemic, and rate their current psychosocial stressors (listed on Table A1) on a 1–5 Likert scale (never to fairly often). The PSS-10 is a 10-item survey assessing how often the respondent felt stress over the previous month, with 6 positively worded and 4 negatively worded items. The CD-RISC is a 25-item survey designed to assess the level of agreement with statements regarding resiliency features over the previous month, including items related to flexibility, self-efficacy, ability to regulate emotion, optimism, and ability to maintain attention under stress. Children completed the Patient-Reported Outcomes Measurement Information System (PROMIS) Pediatric Standard Form Depressive Symptom 8a v2.0 and the PROMIS Pediatric Standard Form Anxiety 8a v2.0. Caregivers completed the PROMIS Standard Form Depression 8a v1.0 and PROMIS Standard Form Anxiety 8a v1.9 and a modified CD-RISC scale to assess their perception of their child’s resiliency. Once surveys were completed, a chart review was conducted for the previous 365 days to assess for hospitalizations, ER visits, acute clinic visits, frequency of follow up, genotype, and history of stroke.

### 2.4. Statistical Analysis

Descriptive statistics were calculated for demographics and other continuous variables, and frequencies with percentages were provided for categorical variables. Pearson’s correlation analysis was used to assess the association between continuous variables (CD-RISC score, PSS-10 score, PROMIS Anxiety score, PROMIS Depression score). ANOVA analysis was performed to detect the differences in scores between categorical variables (genotype, frequency of hospitalization, ER visits, acute clinic visits, stroke, transplant, number of children in home, number of adults in home). Univariate linear regression was performed to assess the relationships between child’s and caregiver’s CD-RISC and PPS-10 scores. The Wilcoxon signed rank test was used to compare caregiver’s/child’s ratings of how well they managed their SCD before and during the COVID-19 pandemic. The intraclass correlation coefficient (ICC) was calculated to assess the agreement of CD-RISC items between the child’s self-report and the caregiver’s assessment of the child. Data were analyzed with SAS^®^ (SAS Institute Inc., Cary, NC, USA) version 9.4. A *p*-value equal or less than 0.05 was considered significant. Survey data were only included in the analysis if both the caregiver and child completed all surveys.

## 3. Results

### 3.1. Distribution of Resiliency and Stress Scores

**Figure 1:**
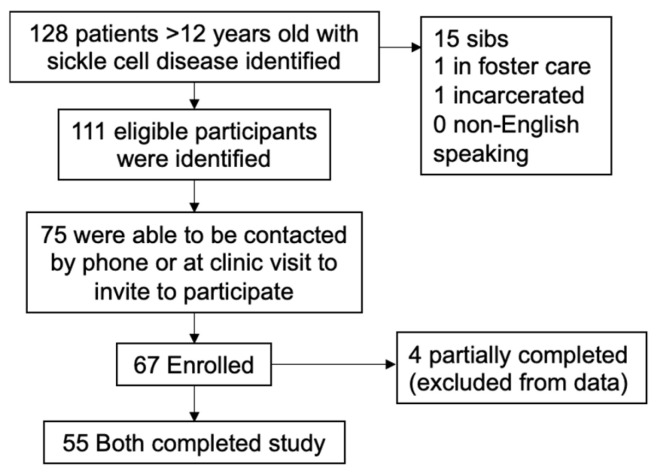
Recruitment and enrollment outcomes: Flow diagram of identified study participants. Inclusion criteria: ≥12 years old, English speaking, and active diagnosis of SCD. Exclusion criteria: child in foster care or sibling enrolled.

A total of 55 child and caregiver dyads were included in this study (Figure 1).

The mean age of the children was 15.2 years (SD = 2.2), while the caregivers’ mean age was 43.4 years (SD = 7.1). Both the median number of adults and children living in the home was 2. All of the caregivers identified as female. The highest level of education completed amongst most caregivers was high school or GED (36.4%) or an associate’s degree (27.3%). Two-thirds of caregivers reported an income of USD 50,000 or less. The majority of children had HbSS (36/55, 65.5%), with the remainder having HbSC (15/55, 27.3%), HbSB+ (1/55, 4.0%), and HbSB0 (3/55, 5.5%). There was a wide range of SCD clinical severity, with 20 (36.4%) children experiencing previous stroke, 4 children (7.3%) with a history of bone marrow transplant, and 14 children (25.5%) with three or more emergency room (ER) visits in the last year. A summary of demographic and clinical information regarding both the caregivers and children in the study is provided in Table 1.

The distribution of caregiver and child resiliency and stress scores are shown in Table 2.

The mean caregiver CD-RISC score was 75.8 (SD = 12.7), which is within the 50th percentile of the general American adult population [19]. Comparatively, the mean child CD-RISC score was 68.5 (SD = 13.3). Although lower than their caregivers’ mean score, child CD-RISC scores were similar to previously reported scores for children with other chronic conditions [14]. Child CD-RISC was significantly positively correlated with age (*r* = 0.28, *p* = 0.04). The average perceived stress scores in both the child and caregiver cohorts were similar (16.1, SD = 6 and 15.3, SD = 7.1, respectively) and were slightly higher compared to a mean of 14.2 in the general adult population [18]. There was no significant correlation between child anxiety and resiliency scores (*r* = −0.14, *p* = 0.31) or child depression and resiliency scores (*r* = −0.25, *p* = 0.07), demonstrating CD-RISC scoring as an independent measure to be used in this cohort to assess resiliency.

### 3.2. Associations Between Stress and Resiliency

**Figure 2:**
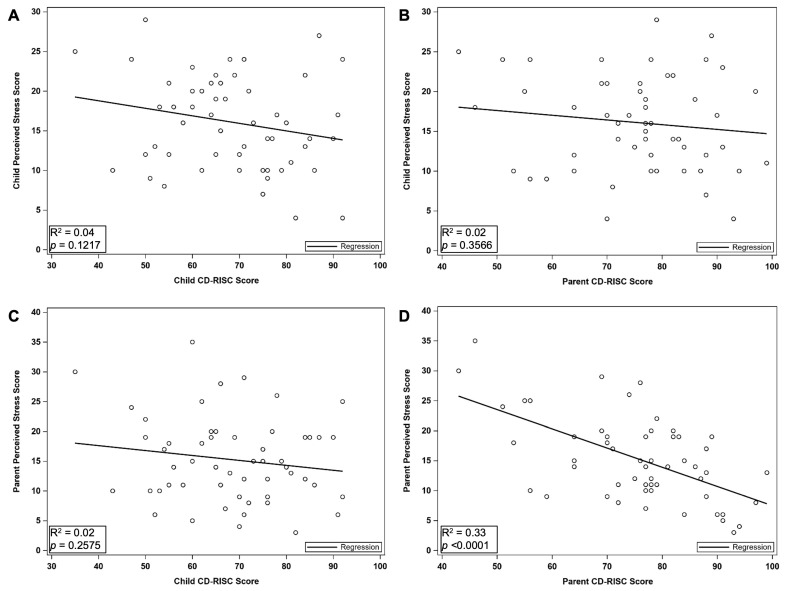
Resiliency and Perceived Stress Scores: (**A**,**B**) There is no association between child resiliency and child perceived stress (*r* = −0.21, *p* = 0.12) or caregiver perceived stress (*r* = −0.16, *p* = 0.26). (**C**,**D**) Caregiver resiliency was not associated with child perceived stress (*r* = −0.13, *p* = 0.36); however, caregiver stress was significantly correlated with caregiver resiliency (*r* = −0.58, *p* < 0.0001).

Perceived stress scores from both caregivers and children were compared to child resiliency scores. Figure 2 demonstrates the relationships between caregiver and child perceived stress and child resiliency scores. Child resiliency was not associated with child’s perceived stress (*r* = −0.21, *p* = 0.12) or caregiver’s perceived stress (*r* = −0.16, *p* = 0.26). Additionally, there was no association between caregiver resiliency and child perceived stress (*r* = −0.13, *p* = 0.36); however, caregiver stress and caregiver resiliency were significantly correlated (*r* = −0.58, *p* ≤ 0.0001).

Common psychosocial stressors and their frequencies were surveyed in identifying factors that may impact stress. Table A1 (Appendix A) lists all stressors and frequencies that were surveyed. The most frequent stressor for caregivers and children was “concern about (my child’s) sickle cell disease”, with 30.9% and 54.5% of children and caregivers, respectively, rating this stressor as being experienced fairly or very often. Other more frequent stressors endorsed by caregivers were “concern about getting others sick” and “financial difficulties”. Given that this study was conducted during the COVID-19 pandemic, both children and caregivers were surveyed on the frequency of stress from the pandemic. Though 26 caregivers (47.3%) reported concern over COVID-19 fairly often or very often, only 25.5% of children reported concern with the same level of frequency. Furthermore, the majority of caregivers and children (74.0%, 90.7%) reported that the quality of sickle cell disease management remained unchanged prior to and during COVID-19, with no significant differences in perceived quality of care.

### 3.3. Lower Resiliency Scores Are Associated with Increased Hospitalizations, but Not with Other Markers of Severe Clinical Disease

**Figure 3:**
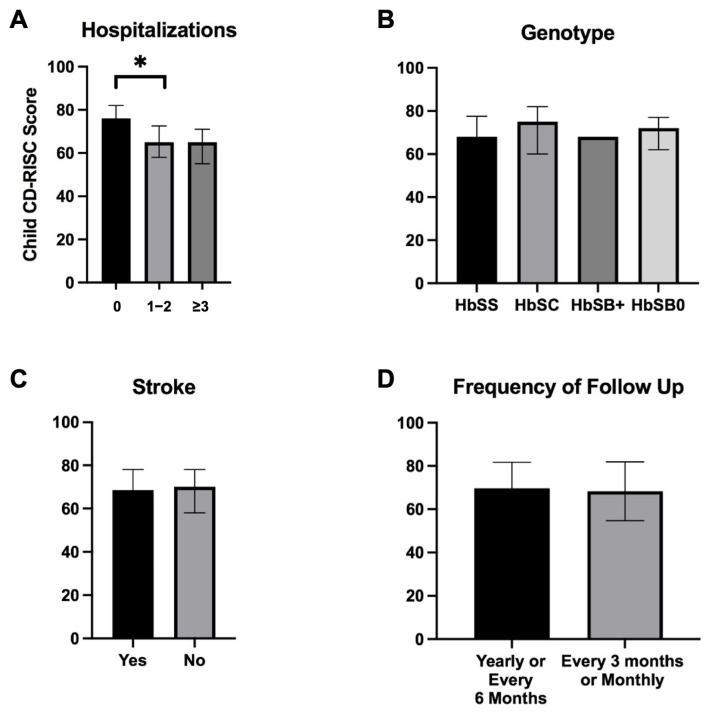
Child Resiliency Scores and Clinical Outcomes: (**A**) Children with one or more hospitalizations had significantly lower resiliency scores than those without hospitalizations within the last year (median 65 vs. 76, *p* = 0.0386). Differences in resiliency scores were not observed in other markers of clinical disease. * indicates *p* < 0.05 (**B**) Resiliency scores were not significantly different between more clinically severe genotypes (HbSS, HbSB0) and clinically mild genotypes (HbSC, HbSB+) (69 vs. 71.5, *p* = 0.80). (**C**) Resiliency scores were not significantly different (68.5 vs. 70, *p* = 0.97) between children who did or did not have a history of stroke. (**D**) Resiliency scores were not significantly different between children who had more frequent follow up (every 3 months or monthly) compared to those with less frequent follow up (yearly or every 6 months) (69.5 vs. 66, *p* = 0.79).

Comparisons with ANOVA testing demonstrate children with one to two hospitalizations in the last year had significantly lower resiliency scores compared to those with no hospitalizations (median 65 vs. 76, *p* = 0.0386; Figure 3). There was no difference in median resiliency scores between children with one to two hospitalizations compared to those with three or more hospitalizations within the last year (Figure 3). Unlike other studies, there was no relationship between child resiliency and ER visits (*p =* 0.89). When comparing resiliency scores across other markers of clinical disease severity, there were no significant differences in median resiliency scores when comparing severe genotypes (HbSS/HbSB0) to milder genotypes (HbSC/HbSB+) (69 vs. 71.5, *p* = 0.80), history of stroke (68.5 vs. 70, *p* = 0.97), history of transplant (65 vs. 70, *p* = 0.76), or frequency of follow up (every 3 months or monthly = 69.5 vs. every 6 months or yearly = 66, *p* = 0.79; Figure 3). To further determine if clinical disease severity impacted hospitalization rates, we compared hospitalization frequency by genotype severity and found no significant differences between severe and mild genotypes (*p* = 0.9309). Child and parent perceived stress scores had no relationship with hospitalizations. There were no significant correlations between genotype, frequency of hospitalizations, history of stroke, history of transplant, or frequency of follow up and caregiver resiliency scores.

### 3.4. Child Resiliency Correlates with Caregiver Resiliency Scores and Can Be Accurately Assessed by Caregiver

**Figure 4:**
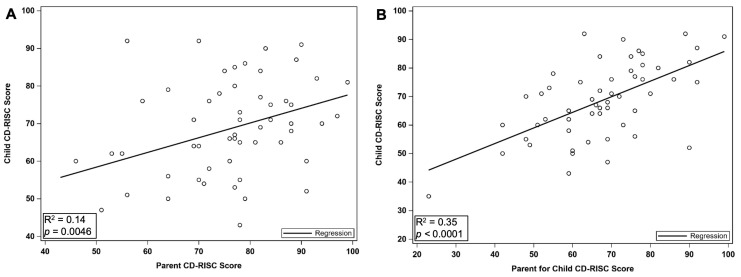
Caregiver Resiliency and Child Resiliency: (**A**) Caregiver resiliency and child resiliency are closely correlated (*r* = 0.38, *p* = 0.0046). (**B**) When caregivers were asked to score their child’s resiliency (caregiver for child), scores were closely correlated with their child’s self-reported resiliency score (*r* = 0.60, *p* < 0.0001).

There was a significant positive correlation between caregiver resiliency and child resiliency (*r* = 0.38, *p* = 0.0046, Figure 4).

Furthermore, when caregivers were asked to assess their child’s resiliency score, univariate analysis showed that caregivers reliably predict their child’s CD-RISC score (+0.55, SE = 0.1, *p* < 0.0001). Though overall CD-RISC scores were significantly correlated between the child and caregiver, 4/25 specific CD-RISC items between the child and caregiver demonstrated significant differences, with caregivers underestimating their child’s rating (Table 3). There were no questionnaire items in which caregivers overestimated their child’s rating.

## 4. Discussion

This study is one of the first to examine resiliency in children with SCD and how it relates to their caregiver’s resiliency and to disease outcomes. Furthermore, it is the first to examine stress in this population during the COVID-19 pandemic. Previous studies have demonstrated the importance of resiliency in chronic illness, and our findings contribute to this body of evidence. While many studies on resiliency focus specifically on ACEs, we measured stress more broadly, as children with SCD face unique challenges relating to vaso-occlusive crises, hospitalizations, and other disease complications. The COVID-19 pandemic also brought about unique challenges for people with chronic illnesses.

We found that while child resiliency and caregiver resiliency were positively correlated, there was not a relationship between perceived stress and resiliency across the groups. We found an inverse relationship between child resiliency scores and hospitalizations that was not present with other markers of clinical disease severity. The most frequently rated concern among both children and caregivers was about their/their child’s SCD, even over concern about COVID-19. Neither group felt there was a significant difference in the management of their SCD during the COVID-19 pandemic.

Pernell et al. showed that higher ACEs are associated with more ER visits for pain but not more hospitalizations for pain in their cohort of children and adolescents with SCD [12]. We found that hospitalizations, but not ER visits, had an association with child resiliency. This could be because we did not directly measure ACEs in this study. Furthermore, our study was limited to one site, and SCD management may vary between providers and hospital sites. If hospitalizations themselves act as ACEs, one would hypothesize that children with more severe phenotypes would have more frequent hospitalizations, leading to lower resiliency. However, this finding was independent of other markers for more severe disease phenotypes including the HbSS and HbSB0 genotypes, history of stroke, or history of transplant. Studies have shown that hospitalization can lead to psychological distress for patients and their caregivers, which can have a lasting impact, with resiliency as a protective factor [20,21,22]. Yet, there is no dose-dependent relationship, as three or more hospitalizations in the last year had the same impact as one to two hospitalizations on resiliency. One potential explanation could be that resiliency impacts how often a child requires hospitalization, rather than hospitalization leading to lower resiliency. To our knowledge, a relationship between hospitalization and resiliency has not previously been described, even with other chronic diseases. In a study of adults with chronic pain, 10-year mortality was significantly better for those with low pain-related disability (defined as resilient), even when there were high chronic pain scores, but there was no difference in days hospitalized [23]. Adults with SCD with high hospital usage and their caregivers have described relationship tensions and fears regarding increasing age and early death [24]. This indicates that interventions to reduce hospitalizations are important to improve quality of life even into adulthood. Our study supports the idea that targeting resiliency could help towards this goal.

Transition to adult care is a well-described period of increased morbidity and mortality for young adults with SCD [25], with previous studies focused on interventions to improve transition readiness [26,27,28]. Higher resiliency is associated with improved transition readiness in adolescents with chronic illness, including SCD [14]; however, there have not been studies focused on improving resiliency for adolescents with SCD. Interestingly, we found that caregiver resiliency was not correlated with any of the children’s disease outcomes, despite being directly correlated with child resiliency. Alternatively, this raises the question about whether child disease outcomes could be improved through increasing caregiver resiliency alone. Targeting caregiver’s resiliency may only be helpful if it also impacts child’s resiliency. Proxy resiliency training is likely insufficient, but interventions to target both child and caregiver resiliency may be more effective than only targeting child resiliency. Others have developed effective training tools for adolescents and caregivers to increase resiliency, but they have not been tested in this population [29,30]. Medical specialty camps may also be a method to improve resiliency for children with SCD [31].

We also contend that early interventions relating to child and caregiver resiliency, rather than waiting for the transition period, would likely be more effective. The CD-RISC scale could be used as a screening tool to identify families that could benefit from resiliency training. The current scale has not been used in children <12 years old. Given that we showed that caregivers are reliable at rating their child’s resiliency, it is possible that parents could score their child’s resiliency in younger age groups, although this has yet to be tested. Others have shown that parents can reliably report their child’s pain, anxiety, and depressive symptoms ~45–55% of the time, but proxy reporting is worse when the caregivers themselves suffer from these symptoms [32].

Others have focused on the importance of psychosocial support for the management of children with SCD [15]. Our study agrees, as resiliency was lower and perceived stress was higher for caregivers who were the only adult in the home. As a source of psychosocial support, children who receive care at a family-centered medical home are more likely to exhibit resilience [3]. We did not assess access to primary care medical homes in our study, but hematology clinics often provide resources and serve a similar function to family-centered medical homes. We did not find a relationship between resiliency and follow-up frequency, suggesting that frequency of healthcare touchpoints does not impact resiliency in our study population.

While others have shown relationships between child–caregiver dyad stress and resiliency [16], we did not find any relationship between stress and resiliency. We intentionally sought to assess caregiver–child dyads in a stable period of child health and did not recruit patients while hospitalized. This relationship may be more evident during a period of higher stress, or a lack of this relationship may reflect that the development of resiliency is a longitudinal process. A qualitative research approach could be used to identify stressors and understand the impact on children and caregivers and to capture cofounding factors. For example, healthcare access, medication adherence, or changes to the support system could be confounding factors that influence both hospitalization and the resiliency of children or caregivers. Others have shown how this approach can be useful in identifying resiliency factors from a caregiver’s perspective [33]. All of our measures also relied on a self-rating scale, which likely introduced reporting biases including social desirability or memory recall.

While PSS-10 scores were slightly higher in our cohort compared to other population studies, they are similar to studies using PSS-10 during the COVID-19 pandemic [34]. These findings suggest that while resiliency impacts those with chronic illnesses uniquely, by affecting their health outcomes, a diagnosis of SCD may not have been the primary driver for the development of resiliency in our cohort. For example, a recent study showed a relationship between neighborhood disadvantage and missed preventative visits but not acute care utilization [35]. Future studies should assess external stressors more broadly, and these potential confounding factors should be considered when assessing stress and resiliency in patients with a chronic illness. It is encouraging that our study does not suggest that the challenges faced by children with SCD lead to lower resiliency overall. The COVID-19 pandemic has led to increased public focus on resiliency and its importance for quality of life for everyone.

This study, a cross-sectional, single-institution study, had limitations and the results should be interpreted with caution. While able to provide preliminary data to demonstrate significant relationships, our sample size did not reach the study’s recruitment goal, further limiting the generalizability of this single-institution study. The ability to capture relationships between stress and resiliency and more subtle nuances between the variables used in this study may have been impacted due to the sample size. Future studies should expand the understanding of resiliency in this population and improve generalizability by adding additional sites to recruit a larger sample size to draw stronger conclusions. Additionally, causal relationships cannot be identified from the cross-sectional design. Future studies should include a longitudinal component to capture how resiliency and its influence on disease outcome change over time and potentially identify confounding variables. There are several confounders of stress and hospitalization outcomes that were not included in this study, such as socio-economic status, caregiver mental health, and access to care. While beyond the scope of this exploratory study, relationships between social determinants of health and caregiver resiliency should be further investigated. Resiliency and stress metrics were based on self-report and thus may have introduced biases. Tools to measure resiliency and stress in this study have previously been validated; however, objective metrics may be useful to incorporate in future studies.

## 5. Conclusions

In summary, child resiliency is linked to hospitalizations in children with SCD. The resiliency of children with SCD and that of their caregivers are related, so interventions to improve resiliency should consider targeting both groups. There is still much to learn about resiliency in children with SCD to improve their health and quality of life.

## Figures and Tables

**Table 1:** Demographics.

	Caregiver *^a^* (N = 55)	Child (N = 55)
**Age (Mean, SD)**	43.4 (7.1)	15.2 (2.2)
**Female Gender (N %)**	55, 100%	24, 43.6%
**Race (N, %)**		
Black or African American	54, 98.2%	53, 98.2%
White	1, 1.8%	0
Other/Prefer not to say/No response	0	2, 3.6%
**Ethnicity (N, %)**		
Hispanic/Latino/Spanish Origin	0	1, 1.8%
Not Hispanic/Latino/Spanish Origin	50, 90.9%	48, 87.3%
No response	5, 9.1%	6, 10.9%
**Number of adults living in home (median, IQR)**	2 (1–2)	
**Number of children living in home (median, IQR)**	2 (1–3)	
**Highest level of completed education (N, %)**		
Some high school	5, 9.1%	
Completed high school or GED	20, 36.4%	
Trade school or associate degree	15, 27.3%	
Bachelor’s degree	8, 14.5%	
Post graduate coursework	6, 10.9%	
Doctorate degree	1, 1.8%	
**Household annual income (N, %)**		
<$10,000	11, 20.0%	
$10,000–25,000	10, 18.2%	
$25,000–50,000	16, 29.1%	
$50,000–75,000	9, 16.3%	
$75,000–100,000	5, 9.1%	
>$100,000	4, 7.3%	
**History of Stroke (N, %)**		20, 36.4%
**History of Transplant (N, %)**		4, 7.2%
**Number of ER Visits in last year (N, %)**		
0		27, 49.1%
1–2		14, 25.5%
≥3		14, 25.5%
**Number of Hospitalizations in last year (N, %)**		
0		26, 47.3%
1–2		16, 29.1%
≥3		13, 23.6%

*^a^* Caregivers identified as primary caretaker living with the child.

**Table 2:** Child and caregiver resiliency and perceived stress scores.

Score	Mean (SD)	Median (IQR)	Range
**Child CD-RISC**	68.5 (13.3)	69 (60–78)	35–92
**Caregiver CD-RISC**	75.8 (12.7)	77 (70–84)	46–99
**Child PSS-10**	16.1 (6)	16 (11–21)	4–29
**Caregiver PSS-10**	15.3 (7.1)	14 (10–19)	3–35
**Caregiver for Child CD-RISC**	67.4 (14.4)	67 (59–76)	23–99

CD-RISC: Connor Davidson-Resilience Scale, PSS-10: Perceived Stress Scale, SD: standard deviation, IQR: interquartile range.

**Table 3:** Discordant CD-RISC items between caregiver for child and child resilience score.

Discordant CD-RISC Items Between Caregiver for Child and Child Resilience Score	(N, %)
**Good or bad, I believe that most things happen for a reason**	*p* = 0.0025
Parent > Child	8 (14.55%)
Parent = Child	22 (40%)
Parent < Child	25 (45.45%)
**I am able to handle unpleasant or painful feelings like sadness, fear, and anger**	*p* = 0.0144
Parent > Child	7 (12.73%)
Parent = Child	30 (54.55%)
Parent < Child	18 (32.73%)
**In dealing with life’s problems, sometimes you have to act on a hunch without knowing why**	*p* = 0.0435
Parent > Child	11 (20%)
Parent = Child	24 (43.64%)
Parent < Child	20 (36.36%)
**I work to attain my goals no matter what roadblocks I encounter along the way**	*p* = 0.0315
Parent > Child	9 (16.36%)
Parent = Child	27 (49.09%)
Parent < Child	19 (34.55%)

## Data Availability

The original contributions presented in this study are included in the article. Further inquiries can be directed to the corresponding author.

## Abbreviations

The following abbreviations are used in this manuscript:

ACE	Adverse childhood experiences
SCD	Sickle cell disease
ER	Emergency room
PSS-10	Perceived stress scale
CD-RISC	Connor Davidson-Resiliency Scale
PROMIS	Patient-Reported Outcome Measurement Information System

## Appendix A

**Table A1:** Frequency of stressors.

Child Stressors	N (%)	Caregiver Stressors	N (%)
**Financial difficulties**		**Financial difficulties**	
Never	28 (50.91%)	Never	4 (7.27%)
Almost Never	11 (20%)	Almost Never	9 (16.36%)
Sometimes	12 (21.82%)	Sometimes	26 (47.27%)
Fairly Often	2 (3.64%)	Fairly Often	9 (16.36%)
Very Often	2 (3.64%)	Very Often	7 (12.73%)
**Difficulty with parent**		**Difficulty with child**	
Never	31 (56.36%)	Never	19 (34.55%)
Almost Never	11 (20%)	Almost Never	15 (27.27%)
Sometimes	10 (18.18%)	Sometimes	17 (30.91%)
Fairly Often	0 (0%)	Fairly Often	2 (3.64%)
Very Often	3 (5.45%)	Very Often	2 (3.64%)
		**Difficulty between my children**	
		Never	24 (43.64%)
		Almost Never	13 (23.64%)
		Sometimes	14 (25.45%)
		Fairly Often	3 (5.45%)
		Very Often	1 (1.82%)
**Difficulty with my extended family *^a^***		**Difficulty with my extended family**	
Never	14 (25.45%)	Never	19 (34.55%)
Almost Never	11 (20.37%)	Almost Never	20 (36.36%)
Sometimes	8 (14.81%)	Sometimes	10 (18.18%)
Fairly Often	1 (1.85%)	Fairly Often	5 (9.09%)
Very Often	1 (1.85%)	Very Often	1 (1.82%)
**Difficulty with my school work**		**Difficulty with work or school work**	
Never	28 (50.91%)	Never	28 (50.91%)
Almost Never	15 (27.27%)	Almost Never	13 (23.64%)
Sometimes	18 (32.73%)	Sometimes	12 (2%)
Fairly Often	5 (9.09%)	Fairly Often	2 (3.64%)
Very Often	3 (5.45%)	Very Often	0 (0%)
**Concern for my physical safety**		**Concern for my physical safety of myself or my Children**	
Never	36 (65.45%)	Never	29 (52.73%)
Almost Never	10 (18.18%)	Almost Never	11 (20%)
Sometimes	9 (16.36%)	Sometimes	10 (18.18%)
Fairly Often	0 (0%)	Fairly Often	5 (9.09%)
Very Often	0 (0%)	Very Often	0 (0%)
**Concern about getting sick from COVID-19**		**Concern about getting sick from COVID-19**	
Never	13 (23.64%)	Never	4 (7.27%)
Almost Never	8 (14.55%)	Almost Never	7 (12.73%)
Sometimes	20 (36.36%)	Sometimes	18 (32.73%)
Fairly Often	7 (12.73%)	Fairly Often	9 (16.36%)
Very Often	7 (12.73%)	Very Often	17 (30.91%)
**Concern about getting others sick**		**Concern about getting others sick**	
Never	27 (49.09%)	Never	15 (27.27%)
Almost Never	10 (18.18%)	Almost Never	9 (16.36%)
Sometimes	13 (23.64%)	Sometimes	12 (21.82%)
Fairly Often	2 (3.64%)	Fairly Often	11 (20%)
Very Often	3 (5.45%)	Very Often	8 (14.55%)
		**Concern about caring for someone who is sick**	
		Never	15 (27.27%)
		Almost Never	12 (21.82%)
		Sometimes	15 (27.27%)
		Fairly Often	7 (12.73%)
		Very Often	6 (10.91%)
**Concern about other health issues**		**Concern about other health issues**	
Never	17 (30.91%)	Never	10 (18.18%)
Almost Never	11 (20%)	Almost Never	10 (18.18%)
Sometimes	15 (27.27%)	Sometimes	25 (45.45%)
Fairly Often	7 (12.73%)	Fairly Often	4 (7.27%)
Very Often	5 (9.09%)	Very Often	6 (10.91%)
**Concern about sickle cell disease**		**Concern about my child’s sickle cell disease**	
Never	13 (23.64%)	Never	2 (3.64%)
Almost Never	7 (12.73%)	Almost Never	8 (14.55%)
Sometimes	18 (32.73%)	Sometimes	15 (27.27%)
Fairly Often	10 (18.18%)	Fairly Often	15 (27.27%)
Very Often	7 (12.73%)	Very Often	15 (27.27%)

*^a^* One participant gave no response.
